# Complex Systems Models in Biology and Medicine: Generic Properties and Applications

**DOI:** 10.1155/2014/580509

**Published:** 2014-06-15

**Authors:** Roberto Serra

**Affiliations:** Department of Physics, Informatics and Mathematics, Modena and Reggio Emilia University, Modena, Italy

Living organisms are complex systems and indeed some concepts of complex systems science, like those of network theory, have been extensively applied in biology and medicine. However, the penetration of other major concepts of complexity theory is still limited. A good example is the dynamical attractor: in spite of the fact that its application to biology was suggested more than 40 years ago and of the fact that there are several beautiful applications, it is still rarely found in models of biological processes and of medical treatment. The penetration of other candidate general concepts that are still more controversial, like for example, that of criticality, is even slower.

On the other hand, complex systems science promises to give novel and important theoretical insights in medicine and biology, including nervous system, immune system, hormonal systems, and metabolic systems, and it can be also very fruitful from an application-oriented perspective. Moreover, while its methods can be applied to the study of well-defined specific cases, they are particularly well-suited to describe “generic” properties, that are common to a wide class of organisms or processes.

The selection procedure for this special issue has been quite rigorous, resulting in a rejection rate of 50%. The papers that have been selected for publication cover some important and intriguing aspects. The paper by Borrotti et al. shows how it is possible to use evolutionary methods to optimize the design of experiments to find new macromolecules with desirable properties, thereby reducing the number of points to be tested. Ghaffarizadeh and colleagues address the fascinating phenomena involved in cell differentiation and present a tool to analyze and visualize the transitions among different cell types, while Weyland et al. explore the role of compartimentalization for polymer sysnthesis, addressing both biological cells and artificial systems. Scheidegger et al. propose a novel model of the response of tumor cells to radiation and heat, amenable to experimental testing, and Dresp-Langley's paper suggests the existence of a universal power law for the adaptive balance of motor responses to environmental stimuli from various sensor modalities. Finally, Sahiner and Yigit show how rough set theory can be applied to medical diagnosis.

While much has still to be done, it is worthwhile to emphasize that, taken together, the papers presented in this special issue of Computational and Mathematical Models in Medicine provide further evidence in favour of the usefulness and importance of complex systems science in medicine.

*Roberto Serra* *Roberto Serra*

